# Molecular targets of neuroplasticity in ischemic stroke: insights from GEO database, single-cell analysis and immune infiltration analysis

**DOI:** 10.3389/fnagi.2025.1561282

**Published:** 2025-07-25

**Authors:** Haoyue Yang, Zekun Li, Lu Zhang, Haifeng Zhang, Yang Liu, Wei Chen, Feng Zhang

**Affiliations:** ^1^Physical Education School, Hebei Normal University, Shijiazhuang, China; ^2^Department of Rehabilitation Medicine, The Third Hospital of Hebei Medical University, Shijiazhuang, China; ^3^Key Laboratory of Measurement and Evaluation in Exercise Bioinformation of Hebei Province, Shijiazhuang, China

**Keywords:** ischemic stroke, GEO analysis, single cells analysis, immune infiltration analysis, neuroplasticity-related genes

## Abstract

This study is aimed to identify diagnostic and therapeutic biomarkers related to neuroplasticity in IS. Gene expression profiling (GSE61616) was derived from GEO, and neuroplasticity-related genes were obtained from the GeneCards databases. The overlapping genes related to neuroplasticity were processed for GO and KEGG analysis. The protein interaction network and hub genes were identified using Cytoscape and the PPI network. Then we predicted the potential TFs and miRNAs related to hub genes. Single-cell analysis was performed to explore cellular localization and intercellular communications related to hub genes in GSE167593. Immune infiltration characteristics were explored via GSVA package. The correlation between various immune cells and hub genes (CCR5 and CXCR4) was calculated via linKET package. Finally, DGIdb database was used for screening small-molecule drugs of CCR5 and CXCR4. Our study screened five significant neuroplasticity-related hub genes (CCR5, CXCR4, TIMP1, GRIN1, and GRM1). Moreover, single-cell analysis revealed that the CCR5 was specifically expressed in microglia and macrophages, while the CXCR4 was specifically expressed in T cells, NK cells, macrophages, and granulocytes. Immune infiltration and correlation analysis revealed a positive association of CCR5 with aDCs and T helper cells, while CXCR4 was positively correlated with CD8+ T cells, but negatively correlated with Tfh. Finally, the Leronlimab, Ulocuplumab, Burixafor, and MSX-122 are promising drugs to treat IS via targeting on CCR5 and CXCR4. In conclusion, our findings suggest that CCR5 and CXCR4 are promising targets for enhancing neuroplasticity post-ischemic stroke, thus providing potentially effective and reliable therapeutic targets for future interventional strategy.

## Introduction

Stroke is the second most common cause of death world-wide, with an estimated one-sixth of the global population experiencing it at least once in their lifetime (Duan et al., 2023; Moskowitz et al., 2010). The number of stroke patients, long-term disability cases and resultant deaths continues to rise each year (Benjamin et al., 2019; Xu et al., 2023). It is speculated that, by the year 2030, the number of stroke-related deaths will reach 12 million, and the number of stroke survivors will increase to 70 million (Feigin et al., 2014). Importantly, stroke-related disability imposes a significant economic, social and emotional burden on both individuals and society (Cornec et al., 2017). In addition, approximately one-third of patients who survive for 6 months after a stroke become dependent on others (Durukan and Tatlisumak, 2007). There are two major types of strokes: ischemic stroke (IS) and hemorrhagic stroke (Chang et al., 2021). IS is the predominant category of strokes, constituting 87% of all stroke cases, which is caused by the blockage of a major cerebral artery (mainly the middle cerebral artery) or its branches due to a thrombotic or embolic event (Barthels and Das, 2020; Durukan and Tatlisumak, 2007; Uzuner and Uzuner, 2023).

Currently, the commonly used medication approved by the Federal Drug Administration for IS is the recombinant tissue plasminogen activator (r-tPA), which is a thrombolytic agent, thus breaking down blood clots and restoring blood flow to the brain (Catanese et al., 2017; Heckman et al., 2023; Nagamine, 2023; Uzuner and Uzuner, 2023; Yawoot et al., 2021). Nevertheless, not every patient experiencing IS is suitable for the medication, because r-tPA needed to be administered within 4.5 h after the beginning of ischemia to reduce the likelihood of hemorrhage (Heckman et al., 2023; Yang et al., 2023; Yawoot et al., 2021). In fact, administering r-tPA beyond this time frame leads to detrimental side effects, such as hemorrhagic transformation (HT), edema, and neurotoxicity, which can contribute to high mortality in stroke patients (Figueroa et al., 2021; Jickling et al., 2014). And only a small percentage of stroke patients, ranging from 5 to 20%, are eligible for r-tPA treatment (Figueroa et al., 2021). Currently, the definitive treatment for IS is limited and most of existing therapies only provide symptomatic relief (Mikitsh and Chacko, 2014). Hence, there is an urgent need for the development of novel and more effective therapeutic approaches (Gribkoff and Kaczmarek, 2017).

In this study, we use Gene Expression Omnibus (GEO) database, single-cell analysis and immune infiltration analysis to uncover novel insights into the mechanisms of IS (Riis et al., 2023). Based on the above mentioned three analysis methods, our aim is to establish a basis for improving the diagnosis and remedies of IS, with the overarching target of alleviating the impact of IS (Riis et al., 2023).

## Methods

### Datasets and data preprocessing

**Figure 1:**
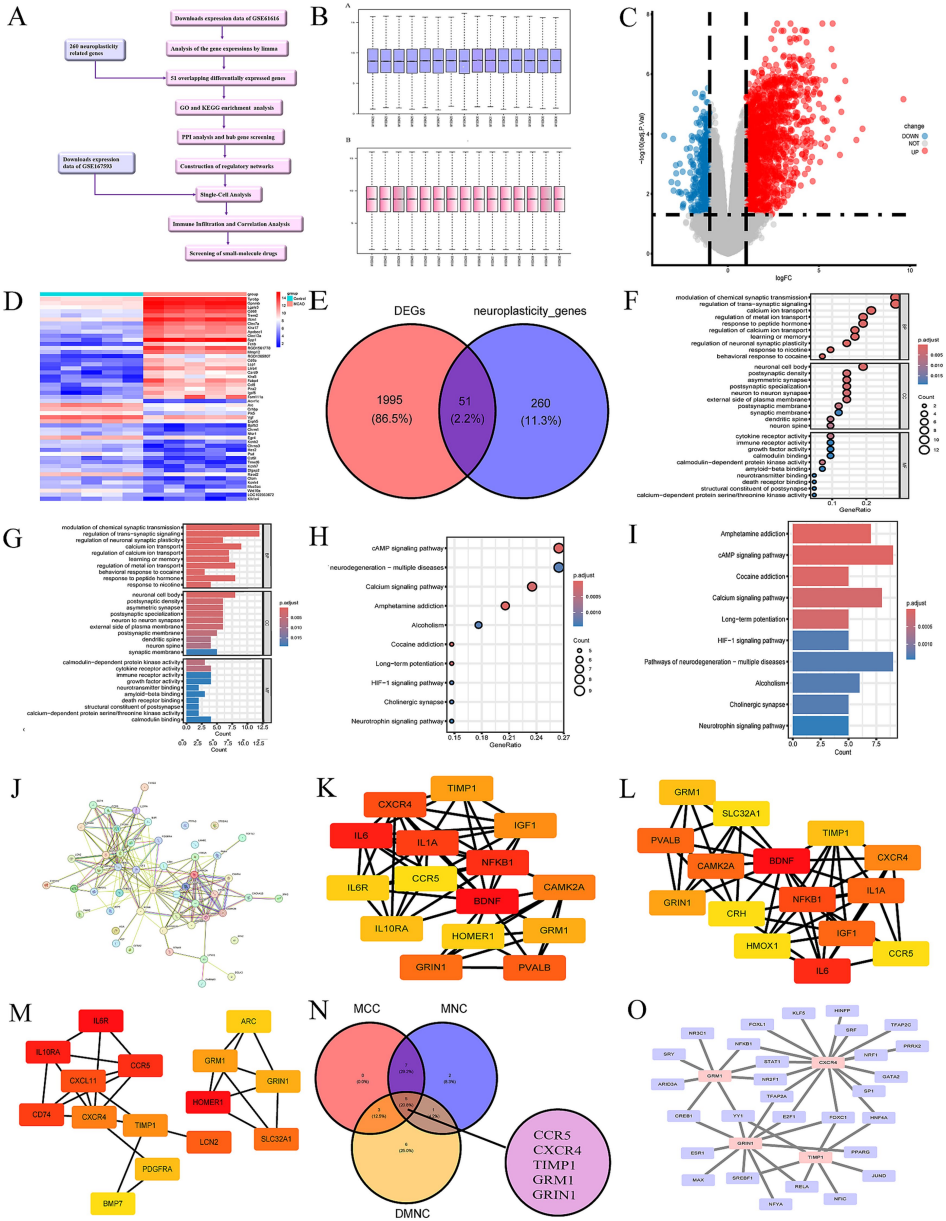
**(A)** The workflow chart. **(B)** Normalization of the expression data in GSE61616. **(C)** Volcano plot of DEGs in GSE61616. Red plot points represent upregulated DEGs, and blue plot points show downregulated DEGs. **(D)** Heatmaps of DEGs in GSE61616. Heatmap showing the DEGs between MCAO and control group in GSE61616. Red represents upregulated genes, and blue indicates downregulated genes. **(E)** Venn diagram shown the 51 overlaps genes between DEGs in GSE61616 and neuroplasticity-related genes. **(F)** Top 10 bubble chart of BP, CC and MF of GO enrichment analysis. **(G)** Top 10 bar chart of BP, CC and MF of GO enrichment analysis. **(H)** Top 10 bubble chart of KEGG enrichment analysis. **(I)** Top 10 bar chart of KEGG enrichment analysis. **(J)** PPI network of the 51 overlaps genes. **(K–M)** The top 15 hub genes of the PPI network. B = maximal clique centrality (MCC), C = maximum neighborhood component (MNC), D = Density of Maximum Neighborhood Component (DMNC). The brighter color in (F, G, and H), the higher score. **(N)** Venn diagram shown the 5 hub genes between MCC, MNC, and DMNC. **(O)** The TFs regulatory networks of the hub genes.

Gene expression profiling datasets and Single-cell transcriptome datasets (GSE61616 and GSE167593) in this study were downloaded from the GEO database^1^ (Ke et al., 2024; Yuan et al., 2024). The datasets were obtained on the basis of the brain tissues of control mice and model mice exposed to MCAO. GSE61616 included 5 ischemic stroke samples and 5 controls using the GPL1355 platform (Table 1). GSE167593 included 1 ischemic stroke sample and 1 control using the GPL24247 platform (Table 1). The workflow chart was demonstrated in Figure 1A.

**Table 1:** Basic information of gene expression profiling in GSE61616 and GSE167593.

GEO accession ID	Platform	Samples (total number)	Number of cases	Number of controls	Country	Year	Author
GSE61616	GPL1355	*Rattus norvegicus* (15)	5 ischemic stroke samples	5 controls	China	2014	Wang L
GSE167593	GPL24247	*Mus musculus* (3)	1 ischemic stroke samples	1 control	China	2021	Shi X

### Identification of differentially expressed genes (DEGs)

To identify DEGs in mouse brain samples from MCAO and control mice in the GSE61616 datasets, we conducted differential expression analysis using the “limma” package (Alhussaini et al., 2024). After batch correction, we established |log2 fold change (FC)| > 1 and adjusted *p*-value < 0.05 as the thresholds.

### Gene ontology (GO) and Kyoto encyclopedia of genes and genomes (KEGG) enrichment analysis

The biological functions of the overlapping genes were analyzed through enrichment analysis of the GO and KEGG. GO and KEGG enrichment analysis were conducted using the R package “clusterProfiler” (Zou et al., 2019). GO enrichment analysis was a common bioinformatics means for exploring widely information in large genetic datasets, encompassing biological processes (BPs), molecular functions (MFs), and cellular components (CCs)(Subramanian et al., 2005). Furthermore, KEGG pathway enrichment analysis was usually applied to gain insights into biological mechanisms and functions of the overlapping genes (Kanehisa et al., 2019).

### Protein–protein interaction (PPI) network analysis and hub gene screening

The STRING database^2^ was utilized to build the PPI network, and visualization was performed with Cytoscape software version (3.10.1) (Chi et al., 2022).

### Construction of regulatory networks

Transcription factors (TFs) and microRNAs (miRNAs) are the main regulatory factors that govern gene expression (Alhussaini et al., 2024). They play a pivotal role in both the establishment and maintenance of gene expression and epigenetic regulatory frameworks, highlighting their therapeutic potential as targets for treating IS injury (Alhussaini et al., 2024).

NetworkAnalyst database^3^ was utilized to predict regulatory networks of potential TFs based on hub genes (Xia et al., 2015). The multiMiR package and mirtarbase database were employed to predict miRNAs associated with these hub genes. Subsequently, Cytoscape software (3.10.1) was used for further visualization (Chi et al., 2022).

### Single-cell analysis

Single-cell analysis was utilized to validate and evaluate the expression of hub genes (CCR5, CXCR4, TIMP1, GRIN1 and GRM1) at the single-cell level (Perera et al., 2021). Quality control, dimensional reduction, and clustering of the data from the mouse brain datasets (GSE167593) were conducted using Seurat (v.4.0.4) according to a previous paper (Perera et al., 2021). Clusters were annotated using singleR (v.1.0) and corrected with CellMarker (Aran et al., 2019; Zhang et al., 2019). Then the CellChat package was used to evaluated cell–cell communications and significant pathways related to hub genes (Jin et al., 2021).

### Immune infiltration and correlation analysis

We analyzed the immune infiltration characteristics between MCAO and controls group using readxl and GSVA package (Liu et al., 2022). And the correlation between various immune cells and hub genes (CCR5 and CXCR4) expression was calculated by Spearman analysis, via psych, reshape2 and linKET package (Liu et al., 2022). *p* < 0.05 was considered statistically significant.

### Screening drugs

DGIdb database^4^ is a biological application database for screening of drugs, which can be used to screen drugs with high correlation to the disease genes (Tica et al., 2018). Then, we predict several drugs that may reverse the altered expression of CCR5 and CXCR4 (Zhu et al., 2020).

### Molecular docking

CCR5 and CXCR were selected, and their structures were comprehensively characterized through the UniProt website. The structures of the compounds were downloaded from the Pubchem website, and molecular docking was conducted using CB-DOCK2. Subsequently, top-ranked complex conformations in terms of docking scores were selected for visualization, and the visualization part was provided by Pymol software.

## Results

### Data preprocessing

R software (version 3.5.1) was used to perform the bioinformatics analysis (Pearson, 2019). The “affy” package in R was implemented to perform the normalization and background correction of data (Figure 1B). We downloaded a series of matrix flies and corresponding annotation documents from the GEO database. Subsequently, the probe data was correlated to the corresponding genes by the Bioconductor package in R software (Gentleman et al., 2004). In cases where a gene matched with multiple probes, the mean expression value was selected for subsequent analysis (Gentleman et al., 2004). As shown in Table 2, 10 samples (GSM1509422, GSM1509423, GSM1509424, GSM1509425, GSM1509426 GSM1509427, GSM1509428, GSM1509429, GSM1509430, and GSM1509431) were used for subsequent analysis (Table 2).

**Table 2:** Grouping results of gene expression profiling.

ID	Group
GSM1509422	Control
GSM1509423	Control
GSM1509424	Control
GSM1509425	Control
GSM1509426	Control
GSM1509427	MCAO
GSM1509428	MCAO
GSM1509429	MCAO
GSM1509430	MCAO
GSM1509431	MCAO

### Identification of DEGs

The analysis of DEGs (GSE61616) was performed using the “limma” package, obtaining 2046 DEGs (1,533 up-regulated and 513 downregulated). The DEGs presented in the form of a volcano map, and the red parts stands for upregulation and the blue parts stands for downregulation (Figure 1C). The heatmap of 25 most up-regulated and 25 most down-regulated DEGs were shown in Figure 1D. The areas highlighted in red indicate the genes of upregulation, while the sections shown in blue represent the genes of downregulation.

The GeneCards database,^5^ a comprehensive database for human gene search and prediction, was used to obtain 418 genes related to neuroplasticity. A total of 311 neuroplasticity-related genes were obtained after human-mouse homologous gene conversion using the “homologene” package. Among the 311 neuroplasticity-related genes, 51 genes overlapped with DEGs (Figure 1E). Here, we selected the 51 overlapping genes for subsequent analysis.

### GO and KEGG enrichment analyses

GO and KEGG enrichment analysis were used to analyze the potential functions and associated pathways of the 51 overlapping genes. And the top 10 enrichment results were demonstrated in the form of bar charts and bubble charts (Figures 1F–I). The GO analysis clearly suggested that the 51 overlapping genes in the BP were most enriched in “modulation of chemical synaptic transmission” and “regulation of trans−synaptic signaling.” These the 51 overlapping genes in CC were most enriched “neuronal cell body.” Alterations in MF were dominantly brimming with “cytokine receptor activity.” Furthermore, the KEGG analysis highlighted that these genes were mainly participated in the cAMP and calcium signaling pathway.

### PPI network analysis and hub gene screening

The STRING database was used to analyze protein prediction and experimental interactions (Liu et al., 2023). The interactions among 51 overlapping genes were studied by constructing and optimizing a PPI network through the STRING database (Figure 1J). Subsequently, the 5 hub genes (CCR5, CXCR4, TIMP1, GRIN1, and GRM1) were identified by intersecting the results from the three algorithms of CytoHubba (the plugin of Cytoscape) including maximum clique centrality (MCC) (Figure 1K), maximum neighborhood component (MNC) (Figure 1L), and density of maximum neighborhood component (DMNC) (Figures 1M,N).

### Construction of regulatory networks

**Figure 2:**
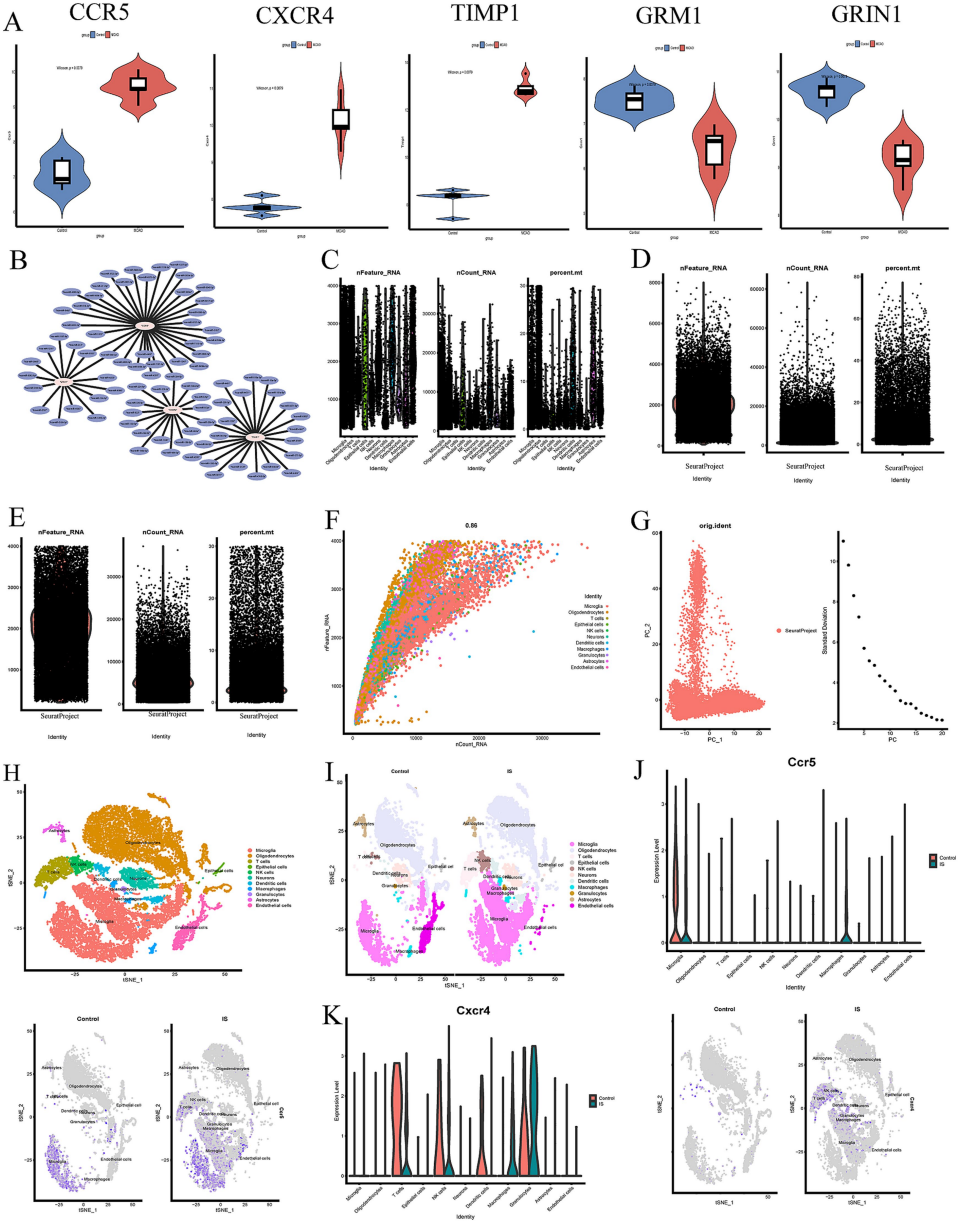
**(A)** Validation of hub genes between MCAO and control sample in violin diagram. **(B)** The miRNAs regulatory networks of the hub genes. **(C–F)** Data filtering process of GSE167593 database. **(G)** PC plot showing linear dimensionality reduction process of hypervariable genes. **(H)** t-SNE plot visualizing clustering of single cells colored by cell types. **(I)** Visualisation of clustering and annotation in the tSNE plot control of MCAO groups and control groups. **(J,K)** The expression of hub genes (CCR5 and CXCR4) of MCAO and controls groups in different cell clusters.

Furthermore, we screened potential TFs and miRNAs that may regulate the hub genes, as illustrated in Figures 1O, 2B. In this study, we identified a total of 29 TFs and 86 miRNAs. Specifically, we predicted 29 target TFs based on the four hub genes (CXCR4, GRIN1, TIMP1, and GRM1) (Figure 1O) and 86 target miRNAs associated with the four hub genes (CCR5, CXCR4, TIMP1, and GRM1) (Figure 2B). These TFs and miRNAs may have a potentially crucial role in the post-ischemic stroke neuroplasticity.

And then, a comparison between MCAO and control groups was assessed to evaluate the expression levels of hub genes (CCR5, CXCR4, TIMP1, GRIN1, and GRM1) (Figure 2A). The results showed that CCR5, CXCR4 and TIMP1 were up-regulated in the MCAO groups compared with control groups, while GRM1 and GRIN1 were down-regulated in the MCAO groups compared with control groups. All these above mentioned results were helpful to better understand the role of neuroplasticity in IS and screen the feasible targets for post-ischemic stroke neuroplasticity.

### Single-cell analysis

**Figure 3:**
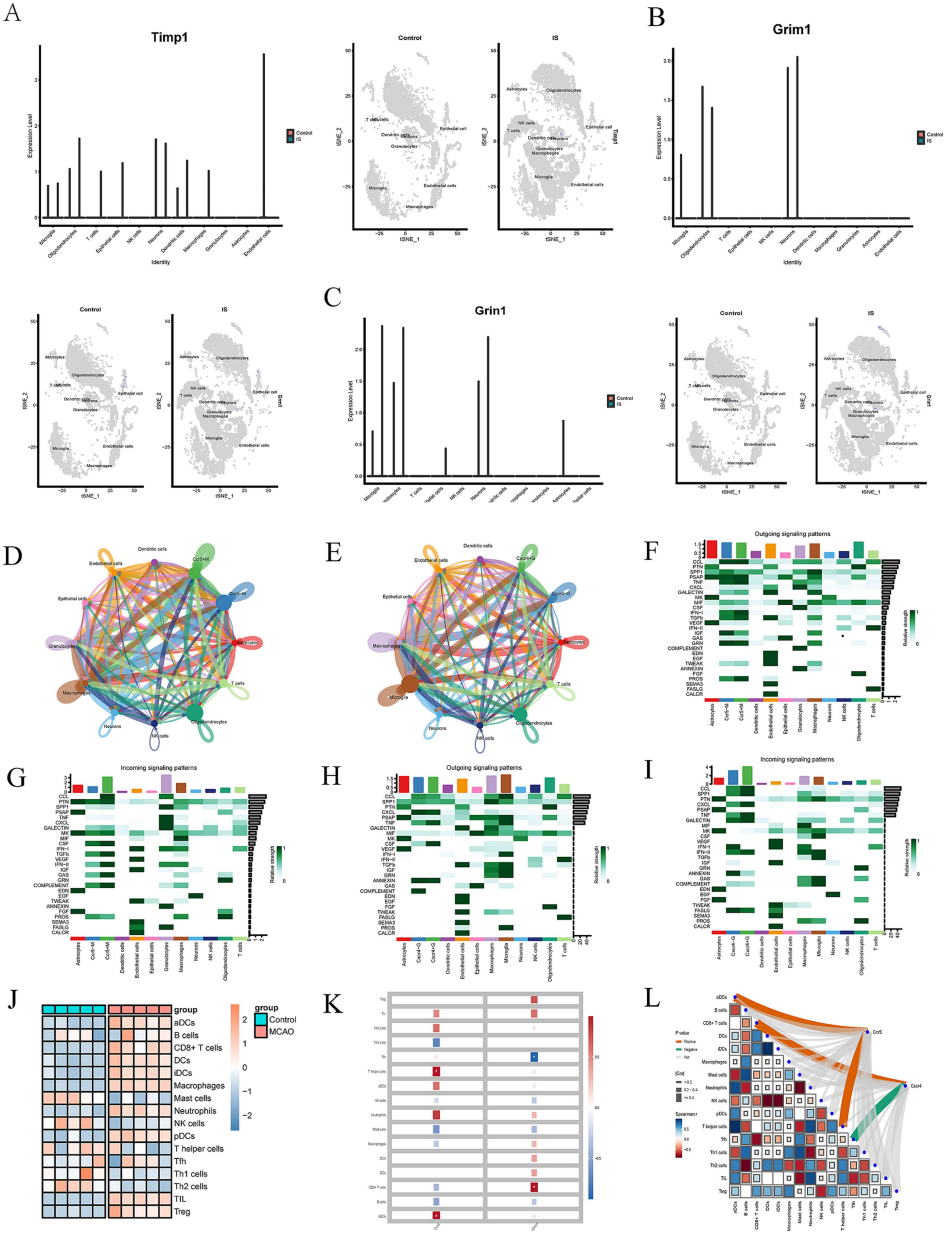
**(A–C)** The expression of hub genes (TIMP1, GRM1 and GRIN1) of MCAO and controls groups in different cell clusters. **(D,E)** An overview of cell–cell interactions. Arrow and edge color indicate direction. Edge thickness indicates the relationship between cells. **(F–I)** Identification of major signaling changes in mela Ccr5 and Cxcr4. Heatmap shows outgoing **(F,H)** and incoming **(G,J)** signaling patterns of Ccr5 and Cxcr4. **(J)** Heatmap depicting the mean differences in the expression of immune-related cells between the MCAO and control groups in GSE61616. Red indicates upregulation, while blue indicates downregulation. **(K,L)** Correlation between Immune Infiltration and Expression of Ccr5 and Cxcr4 in MCAO (*p < 0.05).

Single-cell analysis was performed to explore the cell localization of 5 hub genes (CCR5, CXCR4, TIMP1, GRIN1, and GRM1). The processes of dimensional reduction and cluster annotation of GSE167593 datasets were detailed in Figures 2C–F. To ensure datasets purity and accuracy, measures were taken to remove doublets from each sample, and cell clusters were annotated using SingleR package (Fernandez et al., 2019). A total of 11 cell clusters were identified through cell clustering and annotation (Figures 2G–I). Further analysis of hub genes in cell subpopulations revealed specific expression were illustrated in Figures 2J,K, 3A–C. The results showed that the CCR5 was specifically expressed in microglia and macrophages (Figure 2J), while the CXCR4 was specifically expressed in T cells, natural killer (NK) cells, macrophages, and granulocytes (Figure 2K). Figures 3D,E showed the aggregated cell–cell communication network, and the thickness of connecting lines indicated interaction strength and significant changes in cell types, suggesting the interaction strength and the cell types with significant changes.

Heatmaps were used to identify differential interactions, revealing complex cell–cell networks involving CCR5, CXCR4, and immune cells. Outgoing and incoming signaling patterns of CCR5 and CXCR4 pathways were highlighted in Figures 3F–I, providing insights into specific cell types. Several outgoing signaling patterns of CCR5, including CCL, PSAP, TNF, IFN-I, GRN, and PROS pathways, were exhibited in Figure 3F. Several incoming signaling patterns of CCR5, including CCL, PTN, CSF, TGF-*β*, INF-II, CAS, and COMPLEMENT, were exhibited in Figure 3G. Several outgoing signaling patterns of CXCR4, including CCL, SPP1, CXCL, TNF, CALECTIN and INF-II, were exhibited in Figure 3H. Several outgoing signaling patterns of CCR5, including CXCL, TNF, CSF and ANNEXIN, were exhibited in Figure 3I. These findings demonstrate the single-cell level specificity of CCR5 and CXCR4 in the pathways, guiding future in-depth studies targeting on specific cells.

### Immune infiltration and correlation analysis

Moreover, we investigated the differences in immune-related signatures in the mouse brain between the MCAO and control groups. The heat map intuitively displayed the screening results (Figure 3J). And red indicates upregulation, while blue indicates downregulation. The expression of immune-related signature signatures, including activated dendritic cells (aDCs), CD8+ cells, dendritic cells (DCs), interdigitating dendritic cells (iDCs), macrophages, neutrophils, plasmacytoid DCs (pDCs), T follicular helper (Tfh) cells, tumor infiltrating lymphocytes (TIL) and regulatory T cells (Treg) in the MCAO group were significantly higher than control group.

Subsequently, we explored the correlation between immune infiltration and gene expression of CCR5 and CXCR4 in MCAO. The results indicated there was a positive correlation between CCR5 and aDCs/T helper cells, while CXCR4 was positively correlated with CD8+ T cells, but negatively correlated with Tfh (Figures 3K,L). These findings demonstrated the immune-related signatures of CCR5 and CXCR4, guiding future in-depth studies targeting on specific cells.

### Screening drugs

The DGIdb database was used to screen the potential drugs of CCR5 and CXCR4. In this study, we predicted a total of 13 drugs targeted to CCR5, and eight drugs targeted to CXCR4. Detailed information on drugs was demonstrated in Tables 3, 4. Among them, the drug with the highest correlation scores with CCR5 was Leronlimab, and the highest correlation scores with CXCR4 was Ulocuplumab, Burixafor and MSX-122.

**Table 3:** Small molecule drugs targeting CCR5.

Gene	Drug	Regulatory approval	Indication	Interaction score	Type and directionality
CCR5	LERONLIMAB (C137824)	Not approved	HIV and antiviral agent	7.87	Inhibitor
CCR5	PF-232798	Not approved		5.25	Inhibitor
CCR5	TAK-220 (800)	Not approved		3.93	Inhibitor
CCR5	MARAVIROC (620216)	Approved	HIV and antiviral agent	3.93	Inhibitor
CCR5	BMS-813160 (DB16240)	Not approved		2.62	Inhibitor
CCR5	INCB-9471 (DB12960)	Not approved		2.62	Inhibitor
CCR5	HGS-1025 (CHEMBL2109342)	Not approved		2.62	Inhibitor
CCR5	VICRIVIROC MALEATE (C73146)	Not approved		2.62	Inhibitor
CCR5	CCR5MAB004 (CHEMBL2109341)	Not approved		2.62	Inhibitor
CCR5	VICRIVIROC (C73589)	Not approved	HIV and antiviral agent	2.62	Inhibitor
CCR5	AZD5672 (7686)	Not approved		1.31	Inhibitor
CCR5	PF-04634817 (DB14955)	Not approved		1.31	Inhibitor
CCR5	APLAVIROC HYDROCHLORIDE (C76492)	Not approved		1.31	Inhibitor

**Table 4:** Small molecule drugs targeting CXCR4.

Gene	Drug	Regulatory approval	Indication	Interaction score	Type and directionality
CXCR4	BURIXAFOR (C88323)	Not approved	Adjuvant to stem cell transplantation	6.3	Inhibitor
CXCR4	ULOCUPLUMAB (C95755)	Not approved	antineoplastic agent	6.3	Inhibitor
CXCR4	MSX-122 (DB12715)	Not approved	antineoplastic agent	6.3	Inhibitor
CXCR4	MAVORIXAFOR (C126660)	Not approved	HIV and antiviral agent	5.25	Inhibitor
CXCR4	PLERIXAFOR (733003)	Approved	antineoplastic agent	3.85	Inhibitor
CXCR4	MOTIXAFORTIDE (2664896)	Approved	antineoplastic agent	3.15	Inhibitor
CXCR4	BALIXAFORTIDE (C91094)	Not approved		2.1	Inhibitor
CXCR4	BEVACIZUMAB-AWWB (2046138)	Approved	antineoplastic agent	0.1	Inhibitor

### Molecular docking

Based on the drug prediction results of CCR5 and CXCR4, we selected the highest correlation scores drugs (Leronlimab and Ulocuplumab/Burixafor/MSX-122) for molecular docking. However, there were no available structure of Leronlimab and Ulocuplumab in Pubchem website.

**Figure 4:**
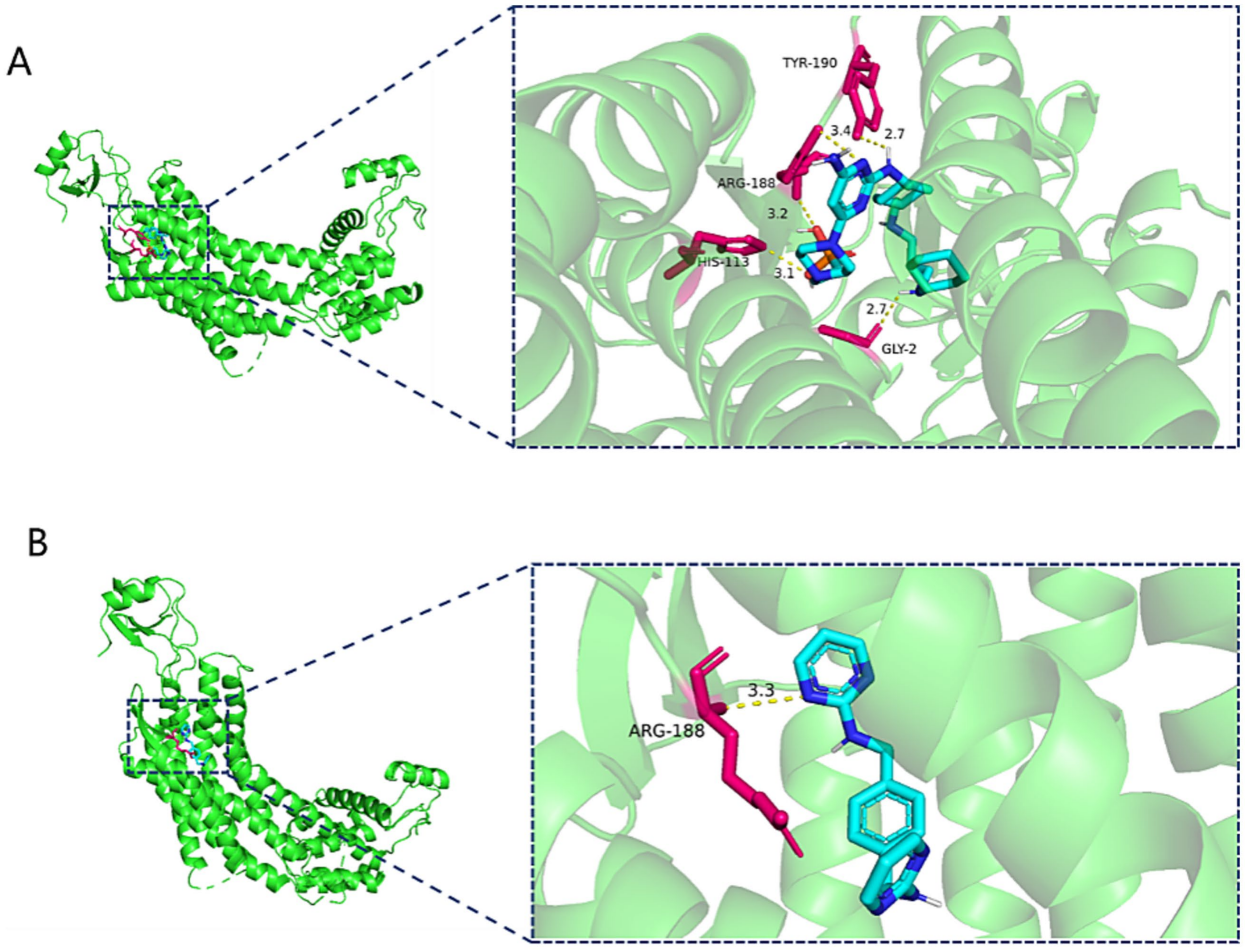
**(A)** Three-dimensional structure of Burixafor-CXCR4 molecular docking. **(B)** Three-dimensional structure of MSX-122-CXCR4 molecular docking.

The molecular docking results showed that Burixafor formed an H-bond with residues GLY-2, HIS-113, ARG-188 and TYR190 of CXCR4 with a docking fraction of −8.2 Kcal/mol (Figure 4). And MSX-122 formed an H-bond with residues ARG-188 of CXCR4 with a docking fraction of −7.4 Kcal/mol (Figure 4).

## Discussion

IS is a serious disease with a high occurrence and mortality rate, making it one of the main causes of lifelong dysfunction of adults (Stichling et al., 2020), resulting in a heavy burden on patients’ families and society (Roychoudhury et al., 2023; Stichling et al., 2020). In spite of notable progress in both diagnostic and treatment methods, it is anticipated that the rate of strokes will increase by more than twofold by the year 2050 (Simats and Liesz, 2022). Additionally, the occurrence of long-term disabilities resulting from strokes is likely to increase similarly, influenced by changes in demographics and a growing number of stroke survivors (Dumbrava et al., 2022; Simats and Liesz, 2022). Neuroplasticity is deemed as the foundation for functional restoration and neurological recovery following stroke, encompassing remodeling of dendrites and dendritic spines, axonal sprouting, synapse shaping, and neurogenesis (Qiao et al., 2023). However, the repair process is frequently incomplete due to the limited regenerative capacity of neurons (Chavez et al., 2017). Thus, it is imperative to develop more accessible diagnosis and treatment approaches in addition to the existing techniques to benefit the stroke patients (Cong et al., 2024; Yan et al., 2023). In this study, we explored potential biomarkers of IS, mechanisms of action, and possible targets related to neuroplasticity based on various bioinformatics analysis.

Our study utilized the GEO database, single cell analysis, and immune infiltration analysis to investigate the pathological processes, marker genes of neuroplasticity, and intercellular communications in the mouse brain following ischemic stroke. Finally, DGIdb database was used to identify drugs with high correlation to the hub genes (CCR5 and CXCR4).

GO enrichment analysis was used to detect the 51 overlapping genes and account for their potential biological mechanisms. The results showed that MF were most enriched in “cytokine receptor activity,” BP were most enriched in “modulation of chemical synaptic transmission” and “regulation of trans−synaptic signaling” and CC were most enriched “neuronal cell body.” Simultaneously, the KEGG enrichment analysis indicated that the cAMP and calcium signaling pathway, along with other signaling pathways, played a vital character in the occurrence and progression of IS.

Then, PPI network was performed to determine the hub genes. Five hub genes, including CCR5, CXCR4, Timp1, Grin1, and Grm1, were screened for subsequent and analysis. We also predicted the TFs and miRNAs regulatory networks, by the NetworkAnalysis database, multiMiR package and Cytoscape software, as shown in Figures 1O, 2B.

To further investigated potential cellular cross talk influencing the development and progression of hub genes, our analysis delved into intercellular communications at the single-cell level. Our findings revealed that the CCR5 was specifically expressed in microglia and macrophages, while the CXCR4 was specifically expressed in T cells, natural killer (NK) cells, macrophages, and granulocytes. These results were consistent with previous studies reported in the literature (Friedman-Levi et al., 2021; Liraz-Zaltsman et al., 2021).

CCR5 is a seven-transmembrane G protein-coupled receptor (Zhou et al., 2016). A series of studies demonstrate that inhibiting CCR5 expression in premotor cortical neurons following stroke can reduce astrocyte reactivity and macrophage recruitment, which may help create a beneficial environment for neural repair (Adelson et al., 2012; Barreto et al., 2012; Liraz-Zaltsman et al., 2021).

Yael et al. demonstrated that CCR5 knockout resulted in advanced cognitive abilities, enhanced neural plasticity, greater neuronal growth, and less brain damage in both humans and animal models (Friedman-Levi et al., 2021). Additionally, CCR5 knockdown induces the upregulation of CREB and downstream proteins, such as dual-leucine zipper kinase proteins, in the premotor cortex (Joy et al., 2019). This process may help preserve dendritic spines, promote axonal sprouting in the contralateral cortex, enhance the remapping of damaged sensory and injured motor circuits, as well as stimulate the formation of new connections within these circuits (Joy et al., 2019).

CXCR4 was present in the CNS from early developmental phase to adulthood, being expressed in neurons, astrocytes, microglia, and ependymal cells (Banisadr et al., 2002; Stumm et al., 2002). CXCR4 plays a key role in neuronal plasticity, repair and immunomodulation in the adult brain (Lee et al., 2016). Lee et al. demonstrated that the interaction between insulin-like growth factor-1 receptor (IGF1R) and CXCR4 led to increased migration and differentiation of stem cells, enhanced neovascularization, and the promotion of neurite regeneration (Ciobanu et al., 2017; Lee et al., 2016).

Furthermore, we identified various outgoing signaling patterns of CCR5, such as CCL, PSAP, TNF, TFN-I, GRN, and PROS pathways, as well as incoming signaling patterns including CCL, PTN, CSF, TGF-*β*, INF-II, CAS, and COMPLEMENT. Similarly, CXCR4 displayed several incoming signaling patterns including CCL, SPP1, CXCL, TNF, CALECTIN, and INF-II. Additionally, CXCR4 exhibited outgoing signaling patterns like CXCL, TNF, CSF, and ANNEXIN. These targets signaling patterns offer insights into the CCR5 and CXCR4 pathways within specific cell types.

The immune microenvironment plays a critical role in the pathophysiological progression of stroke (Chamorro et al., 2012). A variety of immune cells can infiltrate the brain parenchyma orderly following an acute stroke (Chen et al., 2023). As the primary immune cells in the CNS, microglia are participated in numerous aspects of neuroplasticity, including neuronal connectivity, axon formation, dendritic spine reorganization, and endogenous neurogenesis (Sandvig et al., 2018; Wang et al., 2022). Additionally, microglia contribute to tissue repair and functional recovery by secreting anti-inflammatory cytokines and growth factors, clearing cellular debris, promoting nerve regeneration, and remodeling synapses (Wang et al., 2022).

In this study, we confirmed the differences in immune-related signatures in the mouse brain between the MCAO and control groups in GSE61616. The consequences demonstrated that MCAO group had higher expression of activated dendritic cells (aDCs), CD8+ cells, dendritic cells (DCs), interdigitating dendritic cells (iDCs), macrophages, neutrophils, plasmacytoid DCs (pDCs), T follicular helper (Tfh) cells, tumor infiltrating lymphocytes (TIL) and regulatory T cells (Treg) than control group.

Subsequently, we explored the correlation between immune infiltration and the gene expression of CCR5 and CXCR4 in MCAO. Surprisingly, there existed obviously positive correlation between CCR5 and 2 immune cells (aDCs and T helper cells), as well as obviously negative correlation between CXCR4 and Tfh, while obviously positive correlation between CXCR4 and CD8+ cells. These exploratory findings will guide us to further comprehend the critical role of CCR5 and CXCR4.

Moreover, based on screening drugs, we demonstrated that the Leronlimab, Ulocuplumab, Burixafor as well as MSX-122 are the key drugs of CCR5 and CXCR4 in the treatment of IS. Leronlimab is a humanized monoclonal antibody that bound CCR5 (Dhody et al., 2018), which has been extensively tested in clinical settings for treating infections caused by the human immunodeficiency virus type 1 (Dhody et al., 2018; Jacobson et al., 2010). Ulocuplumab (BMS-936564) is the pioneering fully human IgG4 monoclonal anti-CXCR4 antibody (Kuhne et al., 2013), which demonstrated to trigger apoptosis in multiple myeloma cell lines that exhibited high CXCR4 expression (Kuhne et al., 2013). Burixafor is a selective antagonist of the CXCR4 (Hsu et al., 2018). Wan et al. demonstrate that burixafor can alleviate cardiac dysfunction following myocardial infarction in a swine heart transplant model (Hsu et al., 2015). MSX-122 is the only orally administered nonpeptide CXCR4 antagonist (Liang et al., 2012). Previous studies indicated that MSX-122 was used to cancer treatment by inhibiting the growth and metastasis of cancer cells (Kajiyama et al., 2007; Liang et al., 2012; Ramsey and McAlpine, 2013).

The molecular docking results showed that Burixafor formed an H-bond with residues GLY-2, HIS-113, ARG-188 and TYR190 of CXCR4 with a docking fraction of −8.2 Kcal/mol. MSX-122 formed an H-bond with residues ARG-188 of CXCR4 with a docking fraction of −7.4 Kcal/mol. However, there were no available structure of Leronlimab and Ulocuplumab in Pubchem website. And we could not accomplish the molecular docking between Leronlimab/Ulocuplumab with CCR5/CXCR4.

After reviewing the relevant literature, however, there are still no researches to explore the relationship between leronlimab/Burixafor/Ulocuplumab/MSX-122 and stroke. Therefore, these drugs are promising medications to treat IS, but the effectiveness and safety of these drugs in clinical applications for stroke therapy require further evaluation.

However, there are some limitations of our study. First, our study only involves two ischemic stroke cohorts, potentially introducing bias in the assessment of the immune microenvironment in the mouse brain. Second our prognostic model is constructed using data from the GEO public database, which may have inherent selection bias. Third, the limited number of samples and lack of racial diversity in our sample pool further constrain the generalizability of our findings. Finally, some of the drugs we screened have not yet been approved for the treatment of ischemic stroke so far and their safety and effectiveness needed to be further evaluated.

## Conclusion

In this study, we utilize GEO datasets, single cell analysis and immune infiltration analysis to screen hub genes closely correlated with neuroplasticity following ischemic stroke. The results demonstrate that there is a close association of CCR5 and CXCR4 with post-ischemic stroke neuroplasticity. Meanwhile, there is a significant upregulation of CCR5 and CXCR4 following IS. Furthermore, immune infiltration characteristics and the targeting drugs to CCR5 and CXCR4 are also identified to provide useful evidence for IS treatment. In conclusion, our study suggests that CCR5 and CXCR4 are potentially effective and reliable targets for enhancing neuroplasticity following IS.

## Data Availability

The original contributions presented in the study are included in the article/supplementary material, further inquiries can be directed to the corresponding author.

## Footnotes

^1^ http://www.ncbi.nlm.nih.gov/geo

^2^ https://cn.string-db.org/

^3^ http://www.networkanalyst.ca

^4^ https://www.dgidb.org

^5^ https://www.genecards.org/
